# Association between the inability to identify particular odors and physical performance, cognitive function, and/or brain atrophy in community-dwelling older adults from the Fukuoka Island City study

**DOI:** 10.1186/s12877-021-02363-y

**Published:** 2021-07-12

**Authors:** Yujiro Kose, Yoichi Hatamoto, Rie Takae, Yuki Tomiga, Jun Yasukata, Takaaki Komiyama, Yasuki Higaki

**Affiliations:** 1 Faculty of Sports and Health Science, Fukuoka University, 8-19-1 Nanakuma, Jonan-ku, Fukuoka, 814-0180 Japan; 2 The Fukuoka University Institute for Physical Activity, Fukuoka University, 8-19-1 Nanakuma, Jonan-ku, Fukuoka, 814-0180 Japan; 3 Department of Nutrition and Metabolism, National Institute of Health and Nutrition, National Institutes of Biomedical Innovation, Health, and Nutrition, 1-23-1 Toyama, Shinjuku-ku, Tokyo, 162-8636 Japan; 4 Faculty of Nursing and Nutrition, University of Nagasaki, Manabino 1-1-1, Nagayo-Cho, Nishisonogi-gun, Nagasaki, 851-2195 Japan; 5 Faculty of Human Sciences, University of East Asia, 2-1 Ichinomiyagakuen-cho, Yamaguchi, 751-8503 Japan; 6 Center for Education in Liberal Arts and Sciences, Osaka University, 1-17 Machikaneyamachou, Toyonaka, Osaka, 560-0043 Japan

**Keywords:** Olfactory dysfunction, Orange, Hippocampus, Entorhinal cortex, Amygdala

## Abstract

**Background:**

Olfactory dysfunction is associated with severe brain atrophy and cognitive impairment in Parkinson’s disease. However, it remains unknown whether an inability to identify particular odors is associated with physical performance, cognitive function, and/or brain atrophy in community-dwelling older adults.

**Methods:**

In this cross-sectional study, 44 community-dwelling older adults were included (14 males, 30 females; mean age: 72.4 ± 5.7 years, range: 63–85 years). The Odor Stick Identification Test for Japanese, consisting of 12 odors, was used to examine olfaction. Subjects also completed physical performance (lower limb function, balance, and gait speed) and cognitive function (global cognition, logical memory, and the Trail Making Tests). Additionally, magnetic resonance imaging was used to investigate brain atrophy in the bilateral medial temporal area (MTA) and whole gray matter using the voxel-based specific regional analysis system for Alzheimer’s disease.

**Results:**

Total olfaction was not significantly associated with physical performance, cognitive function, or brain atrophy. However, MTA atrophy was associated with an inability to identify Japanese orange (B: − 0.293; β: − 0.347; *p* < .05) after adjusting for age and sex (*R*^2^: 0.328; adjusted *R*^2^: 0.277). Subjects who were unable to identify Japanese orange (*n* = 30) had worse MTA atrophy than those who were able to identify Japanese orange (*n* = 14), even after adjusting for covariates (*p* < .05).

**Conclusions:**

Total olfaction was not associated with physical performance, cognitive function, or brain atrophy. However, an inability to identify Japanese orange odor was independently associated with mild MTA atrophy among community-dwelling older adults.

## Introduction

Parkinson’s disease (PD) and [1] Alzheimer’s disease (AD) are well known neurodegenerative diseases. Olfactory dysfunction is a common non-motor symptom in PD. [2] It is also a predictive marker of dementia in PD patients [3], the development of AD [4], and progression from mild cognitive impairment (MCI) and amnestic MCI to dementia and AD dementia, respectively [5]. Olfactory dysfunction is also associated with memory loss in Japanese individuals with MCI [6].

Recently, it has been reported that cholinergic dysfunction is associated with poor gait performance in PD patients [7, 8], as well as with poor physical performance (mobility, balance, fine motor function, and manual dexterity) in community-dwelling older adults, independently of cognitive function [9]. Furthermore, olfactory function can reportedly be used to predict mortality [10].

However, hyposmia is often silent, even when it is severe/advanced. Takeda et al. suggested that PD patients are often not aware of their own hyposmia, and that hyposmia is often overlooked in the clinic unless olfactory tests are performed [11]. Unfortunately, olfactory examinations are not usually included in common medical checkup packages, and are thus not well known in the general population. However, if an inability to identify any common, familiar odor(s) is associated with impaired physical performance, cognitive dysfunction, and/or brain atrophy, this may help older adults to become aware of physical and/or brain dysfunction at an earlier stage. Previous reports of the association between olfaction and physical and cognitive performance in community-dwelling older adults are scarce. Moreover, to our knowledge, it remains unknown whether an inability to identify any particular odor(s) is associated with cognitive impairment, physical dysfunction, or brain atrophy in such adults.

We thus aimed to conduct a cross-sectional study to investigate whether the ability to identify particular odors is associated with physical performance, cognitive function, and/or brain atrophy in community-dwelling older adults.

## Methods

### Study population

The geographic area of this cross-sectional study was “Island City” (Higashi-ku, Fukuoka, Japan; known as *the Fukuoka Island City Study*) [12, 13]. We included 44 physically independent subjects (14 males, 30 females; mean age: 72.4 ± 5.7 years, range: 63–85 years). The measurement period was from July 2015 to August 2015. The study protocol has been previously described by Takae et al. [12, 13]. The study was conducted according to the guidelines of the Declaration of Helsinki, and all procedures involving human participants were approved by the Ethics Committee of Fukuoka University, Japan (approval no. 15–04-02). The purpose, procedures, and risks of the study were explained to each subject. All subjects provided written informed consent before entering into the study.

### Olfaction

We examined smell identification function using the Odor Stick Identification Test for Japanese (OSIT-J; Daiichi Yakuhin Sangyo Co., Ltd., Tokyo, Japan) [14, 15]. The tests included five clusters and 12 odors. The clusters consisted of wood, grass, and herbs (Indian ink, wood, menthol, and Japanese cypress [*hinoki*]), sweet odors (perfume, rose, Japanese orange, and condensed milk), spices (roasted garlic and curry), gas (cooking gas), and excreta (fermented beans/sweaty socks). These odorants are everyday odors that are familiar to the Japanese population. Each odorant consisted of a solid cream enclosed in microcapsules, shaped like a lipstick.

For this test, each subject first received a piece of folded paraffin paper with one of the 12 odorants rubbed onto it. The subject then lifted the paper close to their nose, opened the paper, and sniffed the odorant. Next, the subject was asked to choose from six possible answers; either “unknown”, “not detected”, or one of four possible answers (consisting of the correct odorant and three other odorants). Each subject repeated these experimental procedures until all 12 odorant sticks had been examined. These methods followed those described by Iijima et al. [16].

We assessed olfaction in three ways: 1) total olfaction, which consisted of the number of correct answers (a score of 0–12); 2) tertiles of total olfaction, in which subjects were divided into tertiles by sex (male: T1–T3, female: T1–T3), and then males and females were combined by tertile group; and 3) the ability to identify particular odors. We scored each odor as 0 (incorrect answer) or 1 (correct answer), and a higher score indicated better olfaction.

### Brain atrophy

Magnetic resonance imaging (MRI) was performed using a 3 T system (Magnetom Skyra; Simens, Munich, Germany). Three-dimensional volumetric acquisition of T1-weighted gradient-echo sequences produced a gapless series of thin sagittal sections, using a magnetization preparation with rapid acquisition (inversion time, 800 ms; echo time, 2.92 ms; repetition time, 1800 ms; slice thickness, 1 mm).

We investigated the degree of brain atrophy using voxel-based specific region analysis for AD (VSRAD) Advance 2 software (Eisai, Tokyo, Japan). VSRAD is a voxel-based MRI analysis software system based on SPM8 plus diffeomorphic anatomical registration through exponentiated Lie algebra [17, 18]. VSRAD Advance 2 automatically calculates z-scores for brain atrophy, which reflect the severity of gray matter atrophy in the whole brain compared with an original database consisting of date from 80 healthy volunteers with no memory impairments or cognitive disorders (37 males, 43 females; mean age: 70.4 ± 7.8 years, range: 54–86 years) [18]. The z-score was defined as (control mean − individual value) / (control standard deviation [SD]). We used four VSRAD Advance indicators: (i) severity of average atrophy in the bilateral medial temporal area, including the hippocampus, part of the amygdala, and the entorhinal cortex; and (ii) significant atrophy of gray matter in the whole brain (%). Atrophy of the medial temporal area was judged based on z-scores: 0–1 (low atrophy), 1–2 (mild atrophy), 2–3 (moderate atrophy), and > 3 (severe atrophy). Lower z-scores indicated less atrophy.

### Cognitive function tests

We used four index cognitive function tests to measure global cognitive function, logical memory, and executive function. All tests were performed by a trained staff member with one-to-one interaction.

Global cognition was tested using a simple test battery for AD screening in community-based settings [19]. This test consisted of 15 points in total, and was made up of four category tasks: an immediate memory test, temporal orientation test, three-dimensional visuospatial perception test, and delayed recall test.

Logical memory was assessed using the Wechsler Memory Scale-Revised (WMS-R) Logical Memory Immediate (LM-I) subtest, which has a maximum possible score of 25. For this test, a short story was read aloud to the subject, who was then immediately asked to recall details of the story.

The Trail Making Tests A and B (TMT-A and TMT-B) [20] were used to assess executive function, and particularly processing speed. For these tests, the complete time taken (in seconds) to complete each task was documented. A lower score in these tests indicated a better performance. In the TMT-A, subjects were asked to draw a line between encircled numbers (1, 2, 3, 4 …), whereas in the TMT-B, they alternated between encircled numbers and Japanese letters (1-あ, 2-い …) to assess set-shifting. Faster TMT scores indicated a better performance.

### Physical performance

We performed four index physical performance tests to measure lower limb muscle power, balance, and gait performance. The protocols that were used followed those described by Kimura et al. [21] and Takae et al. [13].

For the chair stand test, subjects sat on a chair (0.43 m high) without armrests. They sat barefoot and with their arms folded. The subjects then performed a sit-to-stand movement five times as fast as possible, and the time from the initial seated position to the final seated position (after completing five standing movements) was measured. A shorter time indicated a better performance.

For the one leg standing (with open eyes) test, the amount of time that the subject was able to balance on one foot with their eyes open was measured using a stopwatch. For this test, subjects were barefoot and placed their hands on their hips. They were instructed not to let their legs touch each other, and were not allowed to move the foot that was standing on the floor.

Preferred and maximal gait speed: subjects walked 10 m (the first 2 m was included as acceleration and the last 2 m as deceleration) at a comfortable pace, and then at their fastest pace. The 6-m walking time was measured using a phototube. The walking time was measured twice for each test and the average was used. We then calculated the walking speed (m/s) from the 6-m times.

### Physical parameters and comorbidities

The height and weight of each subject were measured using a standard stadiometer and scales, respectively. The body mass index (BMI) was calculated as weight (kg)/height (cm)^2^. Symptoms of depression were tested using the Geriatric Depression Scale (GDS) [22]. Data regarding comorbidities (diabetes mellitus, hypertension, and hyperlipidemia) were self-reported. Diabetes mellitus was determined by a self-report of diabetes mellitus, based on the self-report of a doctor’s diagnosis and/or HbA1c level, obtained by finger-stick testing with commercial kits (Mitsubishi Chemical Medience, Tokyo, Japan).

### Statistical analysis

The unpaired *t*-test was used to compare physical parameters, gait speed, cognitive function, brain atrophy, and olfaction, divided by sex and Japanese orange groups.

The chi-squared test was used to compare comorbidities and the accuracy rates of types of odors divided by sex. It was also used to compare comorbidities among the total olfaction tertile groups and Japanese orange groups.

Analysis of variance (ANOVA) was performed to compare olfaction, physical parameters, gait speed, cognitive function, and brain atrophy among the total olfaction tertile groups.

Analysis of covariance (ANCOVA) was performed to compare olfaction, physical parameters, gait speed, cognitive function, and brain atrophy among the total olfaction tertile groups adjusted for age and sex. It was also used to compare physical parameters, physical performance, cognitive function, and brain atrophy among the Japanese orange groups adjusted for age and sex. In addition, ANCOVA was used to examine the effects of independent covariates on atrophy of the medial temporal area among groups in adjusted models (model A: adjusted for age, sex, body mass index, GDS, diabetes mellitus, hyperlipemia, and hypertension; model B: adjusted for model A, global cognition, WMS-R LM-I, TMT-A, and TMT-B; model C: adjusted for model B, chair stand, one leg standing (with open eyes) test, preferred gait speed, and maximal gait speed; model D: adjusted for model C and whole gray matter atrophy).

Multiple regression (stepwise) analysis was performed to analyze whether physical performance, cognitive function, and brain atrophy were associated with the ability to identify particular odors, as independent covariates. This analysis consisted of three steps: step A: forced entry of covariates (age and sex); step B: stepwise entry of covariates (height, weight, body mass index, GDS, diabetes mellitus, hyperlipemia, and hypertension); and step C: stepwise entry of predictor variables (Indian ink, wood, menthol, Japanese cypress, perfume, rose, Japanese orange, condensed milk, roasted garlic, curry, cooking gas, and fermented beans/sweaty socks).

*p* < .05 was considered to represent statistical significance. Analyses were performed using SPSS v26 for Windows (IBM Corp., Armonk, NY).

## Results

### Subject parameters based on sex

The subjects’ physical parameters, comorbidities, physical performance, cognitive function, and brain atrophy divided by sex are shown in Table 1. Height and weight were significantly higher in males than in females (*p* < .05 for both). GDS and hypertension were significantly higher in females than in males (*p* < .05 for both).

**Table 1:** Subject parameters divided by sex

Variables	All *n* = 44	Male *n* = 14	Female *n* = 30	*p*
*Physical parameters, mean ± SD*				
Age, years	72.4 ± 5.7	71.2 ± 5.8	72.9 ± 5.6	.366
Height, cm	153.2 ± 8.3	162.2 ± 5.7	149.0 ± 5.4	**.000**
Weight, kg	53.7 ± 11.1	61.8 ± 11.2	50.0 ± 8.9	**.000**
BMI, kg/m^2^	22.8 ± 3.6	23.4 ± 3.3	22.5 ± 3.8	.445
GDS, score^a^	2.7 ± 2.5	1.8 ± 1.5	3.1 ± 2.7	**.040**
*Comorbidities, n (%)*				
Diabetes mellitus	17 (39)	7 (50)	10 (33)	.290
Hyperlipemia	12 (27)	2 (14)	10 (33)	.186
Hypertension	10 (23)	0 (0)	10 (33)	**.014**
*Physical performance, mean ± SD*				
Five times CS, s^a^	7.2 ± 1.8	7.0 ± 1.5	7.3 ± 1.9	.641
OLS, s	49.0 ± 39.9	56.2 ± 49.6	45.7 ± 34.9	.481
Preferred GS, m/s	1.28 ± 0.20	1.26 ± 0.20	1.29 ± 0.21	.633
Maximal GS, m/s	1.77 ± 0.25	1.86 ± 0.22	1.72 ± 0.25	.081
*Cognitive function, mean ± SD*				
Global cognition, score	14.0 ± 1.5	14.1 ± 1.8	13.9 ± 1.3	.773
WMS-R LM-I, score	8.7 ± 4.1	8.0 ± 4.7	9.1 ± 3.9	.431
TMT-A, s^a^	96.8 ± 35.9	97.4 ± 39.4	96.5 ± 34.8	.940
TMT-B, s^a^	133.6 ± 69.6	134.1 ± 87.2	133.4 ± 61.4	.974
*Brain atrophy, z-score, mean ± SD*				
Medial temporal area^a^	0.77 ± 0.40	0.89 ± 0.47	0.71 ± 0.35	.161
GM in the whole brain^a^	3.66 ± 1.50	4.07 ± 1.95	3.46 ± 1.23	.297

For the physical parameters, physical performance, cognitive function, and brain atrophy, the *p*-value for male vs. female was calculated using an unpaired t-test

For the comorbidities, the *p*-value for male vs. female was calculated using a chi-squared test

*BMI* body mass index, *GDS* Geriatric Depression Scale, *SD* standard deviation, *CS* chair stand, *OLS* one leg standing with open eyes, *GS* gait speed, *TMT* Trail Making Test, *WMS-R LM* Wechsler Memory Scale-Revised Logical Memory Immediate, *GM* gray matter

^a^Lower scores indicate better performance

### Olfaction parameters based on sex

The total olfaction score was not significantly different between males and females (*p* = .178) (Table 2). For the accuracy rate of each odor, the accuracy rates for wood and condensed milk were significantly higher in females than in males (*p* < .05 for both).

**Table 2:** Olfaction parameters divided by sex

Variables	All *n* = 44	Male *n* = 14	Female *n* = 30	*p*
Total score, *mean ± SD*	6.8 ± 3.3	5.9 ± 3.5	7.3 ± 3.1	.178
*Wood, grass, and herb*				
Indian ink	23 (52)	6 (43)	17 (57)	.393
Wood	27 (61)	5 (36)	22 (73)	**.017**
Menthol	23 (52)	7 (50)	16 (53)	.837
Japanese cypress (*hinoki*)	21 (48)	8 (57)	13 (43)	.393
*Sweet*				
Perfume	27 (61)	6 (43)	21 (70)	.085
Rose	26 (59)	7 (50)	19 (63)	.402
Japanese orange	14 (32)	5 (36)	9 (30)	.705
Condensed milk	26 (59)	5 (36)	21 (70)	**.031**
*Spices*				
Roasted garlic	27 (61)	8 (57)	19 (63)	.694
Curry	34 (77)	10 (71)	24 (80)	.527
*Gas*				
Cooking gas	26 (59)	7 (50)	19 (63)	.402
*Excreta*				
Fermented beans/sweaty socks	27 (61)	8 (57)	19 (63)	.694

For the total score, the p-value for male vs. female was calculated using an un-paired t-test

For each of the 12 odors, the p-value for male vs. female was calculated using a chi-squared test

*SD* standard deviation. The total score consists of the number of correct answers for the 12 odors

### Parameters and performance in the total olfaction tertiles

Sex, comorbidities, physical parameters, physical performance, cognitive function, and brain atrophy were not significantly different between the olfaction tertile groups using ANOVA (Table 3). Worse TMT-A and TMT-B scores were associated with worse total olfaction scores (*p* < .05 for the trend score). However, after adjusting for age and sex, there were no significant associations between any variables and the olfaction tertile groups, and no significant trend scores.

**Table 3:** Subject parameters and performance in each total olfaction tertile

	Tertile of total olfaction			Crude		Adjusted for age and sex	
Variables	T1, *n* = 18	T2, *n* = 13	T3, *n* = 13	*p*	Trend	*p*	Trend
Total olfaction score, mean ± SD	3.9 ± 2.7	7.6 ± 1.6	10.2 ± 0.8	**.000**	**.000**		
Sex, female (%)	13 (72)	8 (62)	9 (69)	.816	.820	-	-
*Comorbidities, n (%)*							
Diabetes mellitus	5 (28)	6 (46)	6 (46)	.469	.281	-	-
Hyperlipemia	6 (33)	2 (15)	4 (31)	.512	.798	-	-
Hypertension	4 (22)	4 (31)	2 (15)	.644	.712	-	-
*Physical parameters, mean ± SD*							
Age, years	73.0 ± 6.2	74.4 ± 5.5	69.5 ± 4.0	.069	.082	-	-
Height, cm	153.8 ± 8.9	151.5 ± 9.7	154.2 ± 5.9	.678	.889	.222	.787
Weight, kg	54.1 ± 12.6	52.9 ± 10.5	54.0 ± 10.3	.951	.981	.831	.650
BMI, kg/m^2^	22.8 ± 4.3	22.9 ± 2.8	22.7 ± 3.7	.988	.907	.929	.744
GDS, score^a^	3.1 ± 2.7	3.1 ± 2.8	1.8 ± 1.6	.270	.139	.421	.228
*Physical performance, mean ± SD*							
Five times CS, s^a^	7.8 ± 2.0	6.9 ± 1.5	6.8 ± 1.6	.262	.163	.241	.450
OLS, s	43.9 ± 34.8	41.3 ± 42.2	63.9 ± 43.1	.281	.173	.682	.421
Preferred GS, m/s	1.26 ± 0.22	1.24 ± 0.18	1.36 ± 0.19	.260	.187	.404	.252
Maximal GS, m/s	1.71 ± 0.25	1.76 ± 0.28	1.86 ± 0.19	.222	.086	.457	.220
*Cognitive function, mean ± SD*							
Global cognition, score	13.8 ± 1.7	13.7 ± 1.5	14.5 ± 0.8	.255	.155	.682	.404
WMS-R LM-I, score	7.4 ± 3.9	9.2 ± 4.3	10.1 ± 3.9	.178	.076	.220	.154
TMT-A, s^a^	106.3 ± 41.7	103.5 ± 36.4	76.9 ± 14.5	.054	**.023**	.250	.098
TMT-B, s^a^	160.1 ± 95.6	124.3 ± 39.6	106.1 ± 29.5	.085	**.033**	.112	.145
*Brain atrophy, z-score, mean ± SD*							
Medial temporal area^a^	0.84 ± 0.35	0.79 ± 0.46	0.66 ± 0.40	.459	.223	.682	.533
GM in the whole brain^a^	3.71 ± 1.65	3.78 ± 1.67	3.46 ± 1.17	.850	.653	.984	.873

Crude: analysis of variance (ANOVA). Adjusted age and gender: analysis of covariance (ANCOVA)

*BMI* body mass index, *GDS* Geriatric Depression Scale, *SD* standard deviation, *CS* chair stand, *OLS* one leg standing with open eyes, *GS* gait speed, *TMT* Trail Making Test, *WMS-R LM* Wechsler Memory Scale-Revised Logical Memory Immediate, *GM* gray matter

^a^Lower scores indicate better performance

### Multiple regression (stepwise) analysis for predicting physical performance, cognitive function, and brain atrophy from the identification of different odors

Greater atrophy of the medial temporal area was significantly associated with an inability to identify Japanese orange, even after adjusting for age and sex (*R*^2^: 0.328; adjusted *R*^2^: 0.277; B: − 0.293; β: − 0.347; 95% confidence interval: − 0.515, − 0.071; *p* < .05) (Table 4). However, worse performances in the chair stand, one leg standing (with open eyes) test, preferred or maximal gait speed, global cognition, WMS-R LM-I, TMT-A, TMT-B, and whole gray matter atrophy were not significantly associated with an inability to identify any particular odor after adjusting for age and sex.

**Table 4:** Multiple regression (stepwise) analysis for predicting physical performance, cognitive function, and brain atrophy from the identification of different odors

	[Step A]	[Step B]	[Step C]	Final model status					
Predicted variables	Forced entry	Stepwise	Stepwise	Variables	B	β	95% CI for B	*R*^2^	adj-*R*^2^
*Physical performance*									
Five times chair stand	Age			Age	0.141	0.449	0.055, 0.228*	.304	.252
	Sex			Sex	0.973	0.256	−0.201, 2.15		
		Weight		Weight	0.079	0.488	0.028, 0.130*		
			-	Constant	−8.88		−16.8, −0.97*		
One leg standing with open eyes	Age			Age	−2.90	−0.414	−4.77, −1.03*	.324	.273
	Sex			Sex	−9.90	−0.117	−32.5, 12.7		
		BMI		BMI	−4.64	−0.422	−7.56, −1.71*		
			-	Constant	381.4		222.1, 540.8*		
Preferred gait speed	Age			Age	−0.005	−0.152	−0.016, 0.006	.028	−.019
	Sex			Sex	0.041	0.095	−0.093, 0.175		
		-	-	Constant	1.60		0.796, 2.41*		
Maximal gait speed	Age			Age	−0.014	−0.330	−0.027, −0.002*	.178	.138
	Sex			Sex	−0.115	−0.220	−0.266, 0.036		
		-	-	Constant	2.99		2.09, 3.90*		
*Cognitive function*									
Global cognition	Age			Age	−0.101	−0.397	−0.176, −0.027*	.156	.115
	Sex			Sex	0.033	0.011	−0.870, 0.936		
		-	-	Constant	21.3		15.8, 26.7*		
WMS-R LM-I	Age			Age	−0.163	−0.225	−0.387, 0.060	.064	.019
	Sex			Sex	1.34	0.153	−1.36, 4.04		
		-	-	Constant	18.3		2.06, 34.5*		
TMT-A	Age			Age	2.74	0.434	1.03, 4.45*	.300	.247
	Sex			Sex	−8.19	−0.108	−28.9, 12.5		
		BMI		BMI	−2.95	−0.299	−5.6, −0.30*		
			-	Constant	−105.8		−235.2, 23.6		
TMT-B	Age			Age	5.69	0.465	2.24, 9.15*	.212	.174
	Sex			Sex	−10.3	−0.070	−52.1, 31.4		
		-	-	Constant	−261.1		−512.1, −10.0*		
*Brain atrophy*									
Medial temporal area	Age			Age	0.030	0.424	0.011, 0.048*	.328	.277
	Sex			Sex	−0.248	−0.294	−0.472, −0.024*		
		-	J orange	J orange	−0.293	−0.347	−0.515, −0.071*		
				Constant	−0.863		−2.21, 0.480		
Gray matter in the whole brain	Age			Age	0.052	0.198	−0.029, 0.133	.075	.030
	Sex			Sex	−0.697	−0.219	−1.67, 0.279		
		-	-	Constant	1.05		−4.82, 6.91		

Type of odors inversed 1 (correct answer) or 0 (un-correct answer)

B: unstandardized regression coefficient, β: standardized regression coefficient, CI: confidence interval, adj-R2: adjusted-R2

Step A covariates consisted of age and gender in a forced entry analysis

Step B covariates consisted of height, weight, BMI, Geriatric Depression Scale, diabetes mellitus, hyperlipemia, and hypertension in a stepwise analysis

Step C predictors consisted of types of odors: Indian ink, wood, menthol, Japanese cypress (hinoki), perfume, rose, Japanese orange, roast garlic, curry, cooking gas, fermented beans/sweaty socks, and condensed milk in a stepwise analysis

*BMI* body mass index, *OLS* one leg standing with open eyes, *J orange* Japanese orange, *TMT* Trail Making Test, *WMS-R LM-I* Wechsler Memory Scale-Revised Logical Memory Immediate

*P<0.05 as a predictor in final models

### Parameters and performance divided by the ability to identify Japanese orange

An inability to identify Japanese orange (*n* = 30) was associated with greater atrophy of the medial temporal area compared with the ability to identify Japanese orange (*n* = 14), but was not significantly associated with any other variables (Table 5). This association was significant even after adjusting for age, sex, GDS, comorbidities, cognition, physical performance, and whole gray matter atrophy (*p* < .05).

**Table 5:** Subject parameters divided by the ability to identify Japanese orange odor (correct/incorrect)

Variables	Correct *n* = 14	Incorrect *n* = 30	*p*-value					
			Crude	Adjusted for age and sex	Adjusted models for medial temporal area atrophy			
					Model A	Model B	Model C	Model D
Sex, female (%)	9 (64)	21 (70)	.705	-				
*Comorbidities, n (%)*								
Diabetes mellitus	6 (43)	11 (37)	.694	-				
Hyperlipemia	4 (29)	8 (27)	.895	-				
Hypertension	3 (21)	7 (23)	.888	-				
*Physical parameters, mean ± SD*								
Age, years	72.7 ± 5.7	72.2 ± 5.8	.784	-				
Height, cm	152.3 ± 9.3	153.7 ± 7.9	.605	.255				
Weight, kg	56.2 ± 12.3	52.6 ± 10.5	.319	.315				
BMI, kg/m^2^	24.1 ± 3.9	22.2 ± 3.4	.112	.118				
GDS, score^a^	2.3 ± 2.1	2.9 ± 2.6	.448	.450				
*Physical performance, mean ± SD*								
Five times CS, s^a^	6.9 ± 1.7	7.4 ± 1.9	.456	.378				
OLS, s	50.2 ± 38.2	48.5 ± 41.3	.899	.830				
Preferred GS, m/s	1.21 ± 0.05	1.32 ± 0.04	.103	.124				
Maximal GS, m/s	1.76 ± 0.07	1.77 ± 0.05	.896	.896				
*Cognitive function, mean ± SD*								
Global cognition, score	14.1 ± 1.4	13.9 ± 1.5	.773	.669				
WMS-R LM-I, score	8.5 ± 4.8	8.8 ± 3.9	.806	.898				
TMT-A, s^a^	92.7 ± 34.9	98.7 ± 36.7	.612	.463				
TMT-B, s^a^	121.6 ± 41.6	139.2 ± 79.4	.443	.308				
*Brain atrophy, mean ± SD*								
MTA, z-score^a^	0.59 ± 0.40	0.85 ± 0.37	**.039**	**.011**	**.007**	**.009**	**.022**	**.0498**
GM in the whole brain, %^a^	3.17 ± 1.37	3.89 ± 1.53	.140	.099				

Crude: analysis of variance (ANOVA). Adjusted age and gender: analysis of covariance (ANCOVA)

Crude: un-paired t-test; adjusted models: analysis of covariance (ANCOVA)

Model A: ANCOVA adjusted for age, gender, body mass index, GDS, and comorbidities (diabetes mellitus, hyperlipemia, and hypertension)

Model B: ANCOVA adjusted for model A and cognitive function tests (global cognition, WMS-R LM-I, TMT-A, and TMT-B)

Model C: ANCOVA adjusted for model B and physical performance (five times chair stand, OLS, preferred gait speed, and maximal gait speed)

Model D: ANCOVA adjusted for model C and gray matter atrophy in the whole brain

*BMI* body mass index, *GDS* Geriatric Depression Scale, *SD* standard deviation, *CS* chair stand, *OLS* one leg standing with open eyes, *GS* gait speed, *TMT* Trail Making Test, *WMS-R LM* Wechsler Memory Scale-Revised Logical Memory Immediate, *MTA* medial temporal area, *GM* gray matter

^a^Lower scores indicate better performance

## Discussion

Total olfaction scores were not significantly associated with physical performance, cognitive function, or brain atrophy in the present study. Only the inability to identify Japanese orange was significantly associated with mild atrophy of the medial temporal area among Japanese community-dwelling older adults, independent of physical parameters, physical performance, cognitive function, and atrophy of the whole gray matter.

Several previous studies have suggested that cholinergic dysfunction is associated with poor gait performance in PD patients [7, 8]. However, there have been few previous studies investigating normally aging older adults. Tian et al. used challenging upper and lower extremity motor function tasks to demonstrate that olfactory function is associated with mobility, balance, fine motor function, and manual dexterity, but is independent of cognitive function. In addition, changes in olfactory function may provide insights into the earliest age-related changes in the brain, which may relate to the neural aspects of age-related movement decline [9].

One of our hypotheses was that worse total olfaction would be associated with worse physical performance, cognitive function, and brain atrophy. Surprisingly, however, the present study revealed that the total olfaction score was not associated with physical performance, cognitive function, or brain atrophy. The reasons for the differences between our results and those of Tian et al. [9] are unclear. One possible reason may be because our study subjects (aged 72.4 ± 5.7 years) were younger than those in the study by Tian et al. (aged 77.4 ± 8.5 years); aging is known to be a strong risk factor for olfactory dysfunction [23]. In addition, several previous longitudinal studies have suggested that olfactory function is a predictor of mortality [10], verbal memory [24], perceptual speed, and episodic memory [25] in subjects without dementia. It is therefore reasonable that our study revealed no association between olfaction and physical performance, cognitive function, or brain atrophy in a cross-sectional study among community-dwelling older adults. Additional studies are needed to investigate whether total olfaction scores are associated with physical performance, cognitive function, and/or brain atrophy in a larger number of community-dwelling older subjects, separated into the youngest older adults and the oldest older adults.

Previous studies into the association between olfaction and brain volume have reported a positive correlation between olfactory performance and gray matter volume in the right piriform cortex in early PD patients [26], and in the nucleus accumbens and left parahippocampal gyrus in very mild amnestic patients (aged 77.4 ± 6.8 years) [27]. Furthermore, Bohnen et al. reported that olfactory dysfunction is positively correlated with acetylcholinesterase activity in the hippocampus, amygdala, and neocortex in PD patients [28]. However, although Hagemeier et al. reported that odor identification deficits are associated with lower volumes in the right hippocampus and left amygdala in amnestic MCI patients, and in the bilateral hippocampus and left amygdala in AD, they found no/weak associations between odor identification and gray matter volume in cognitively normal subjects [29]. Hagemeier et al. suggested that the weak association between olfaction, global atrophy, and aging among cognitively normal subjects may indicate that such interactions are augmented by disease processes [29].

Shiga et al. reported that, in 243 Japanese adults (mean age 37.5 years, range 20–62 years), combinations of two odorants in the smell identification test can be used to screen olfactory impairment [30]. They concluded that the combination of Japanese cypress and Indian ink odorants may be useful for detecting individuals with olfactory impairment (total olfaction scores of ≤7) among Japanese adults who are able to correctly identify the curry odorant. Moreover, Shiga et al. demonstrated that, for olfactory impairment screening with a single odorant, Japanese cypress had the highest positive likelihood ratio (5.2; sensitivity, 42.2%; specificity, 91.9%), while Japanese orange had the second-highest positive likelihood ratio (3.1; sensitivity, 11.1%; specificity, 96.5%) and the highest specificity of all odorants.

It is unclear why an inability to identify orange odor might be associated with atrophy in the medial temporal area. The prevalence of olfactory impairment increases with age [31, 32], and is higher in 80- to 97-year-old individuals (62.5%) than in 70- to 79-year-old individuals (29.2%) [32]. In subjects with similar ages (72.4 ± 5.7 years) to those in the present study, Koskine and Tuorila reported that older subjects had a lower sensitivity to yogurt, but not to grape juice odors [33]. Interestingly, sensitivity to orange flavor (which is used commercially to flavor beverages) was reported to be 49 times lower in older subjects than in younger subjects [34]. These findings indicate that there is specificity of olfactory impairments, and suggest that recognizing orange in particular may be related to brain atrophy.

Japanese orange is an everyday, familiar food and odor to the Japanese population. Japanese oranges are smaller than the familiar oranges of most countries, but their flavors are similar. Thus, an inability to identify orange odor might be helpful for the early detection of silent, advanced hyposmia as well as atrophy of the medial temporal area, within both Japanese and non-Japanese populations. However, olfactory identification tests are not generally popular. This study may therefore help to increase the awareness of hyposmia among community-dwelling older adults in their everyday life.

One strength of our study was the novel finding of an association between an inability to identify Japanese orange and mild atrophy of the medial temporal area, including the hippocampus, part of the amygdala, and the entorhinal cortex. Notably, compared with subjects who were able to identify Japanese orange, subjects who were unable to identify Japanese orange had a similar age, sex ratio, comorbidities, physical performance, cognitive function, and atrophy of gray matter in the whole brain. These results suggest that an inability to identify orange odor is independently associated with silent, advanced atrophy of the medial temporal area. The individuals who were unable to identify Japanese orange would not be judged to have a high risk of dementia from the screening of physical performance and/or cognitive function. Therefore, an impairment in the identification of orange odor might be one of the earliest markers (before physical and cognitive dysfunction) of dementia and/or mortality among community-dwelling older adults. In the future, these findings need to be further defined by a longitudinal study, and the responsible pathways should also be explored.

The limitations of this study were as follows: (i) our cross-sectional study was unable to interpret cause and effect; (ii) it was unknown whether subjects had stroke or cardiovascular disease; (iii) it was unknown whether the characteristics of our study subjects reflected those of the general population; (iv) it was unclear whether our sample size was large enough in terms of statistical power; and (v) the association between olfaction and brain atrophy in non-Japanese subjects remains unclear.

## Conclusion

An inability to identify specific odors (orange, in particular) was significantly associated with mild atrophy of the medial temporal area, including the hippocampus, part of the amygdala, and the entorhinal cortex, in community-dwelling older adults.

## Data Availability

We used data collected by our research team (The Fukuoka University Institute for Physical Activity, Fukuoka University). The datasets obtained and/or analysed during the current study are not publicly available and we are planning to publish more papers using the same dataset. The datasets are available from the corresponding author on reasonable request.

## Abbreviations

MTA: Medial temporal area
PD: Parkinson’s disease
AD: Alzheimer’s disease
MCI: Mild cognitive impairment
OSIT-J: The Odor Stick Identification Test for Japanese
MRI: Magnetic resonance imaging
VSRAD: Voxel-based specific region analysis for Alzheimer’s disease
SD: Standard deviation
WMS-R: The Wechsler Memory Scale-Revised
LM-I: Logical Memory Immediate (LM-I)
TMT: The Trail Making Test
BMI: Body mass index
GDS: The Geriatric Depression Scale
ANOVA: Analysis of variance
ANCOVA: Analysis of covariance
